# Sex-Dependence in the Effect of Pharmaceutical Excipients: Polyoxyethylated Solubilising Excipients Increase Oral Drug Bioavailability in Male but Not Female Rats

**DOI:** 10.3390/pharmaceutics11050228

**Published:** 2019-05-10

**Authors:** Yang Mai, Liu Dou, Christine M. Madla, Sudaxshina Murdan, Abdul W. Basit

**Affiliations:** 1 UCL School of Pharmacy, University College London, 29 – 39 Brunswick Square, London WC1N 1AX, UK; maiy6@mail.sysu.edu.cn (Y.M.); liu.dou.14@ucl.ac.uk (L.D.); christine.madla.16@ucl.ac.uk (C.M.M.); s.murdan@ucl.ac.uk (S.M.); 2 School of Pharmaceutical Sciences (Shenzhen), Sun Yat-sen University, Guangzhou 510275, China

**Keywords:** pharmaceutical excipients, solubilizing agents, sex differences, P-glycoprotein, ranitidine, bioavailability, biopharmaceutical classification system (BCS), drug absorption, Polyethoxylated, multidrug resistance protein 1 (MDR1)

## Abstract

It is known that males and females respond differently to medicines and that differences in drug behaviour are due to inter-individual variability and sex specificity. In this work, we have examined the influence of pharmaceutical excipients on drug bioavailability in males and females. Using a rat model, we report that a portfolio of polyoxyethylated solubilising excipients (polyethylene glycol 2000, Cremophor RH 40, Poloxamer 188 and Tween 80) increase ranitidine bioavailability in males but not in females. The in vivo sex and excipient effects were reflected in vitro in intestinal permeability experiments using an Ussing chamber system. The mechanism of such an effect on drug bioavailability is suggested to be due to the interaction between the excipients and the efflux membrane transporter P-glycoprotein (P-gp), whose expression in terms of gene and protein levels were inhibited by the solubilising agents in male but not in female rats. In contrast, the non-polyoxyethylated excipient, Span 20, significantly increased ranitidine bioavailability in both males and females in a non-sex-dependent manner. These findings have significant implications for the use of polyoxyethylated solubilising excipients in drug formulation in light of their sex-specific modulation on the bioavailability of drugs that are P-gp substrates. As such, pharmaceutical research is required to retract from a ‘one size fits all’ approach and to, instead, evaluate the potential impact of the interplay between excipients and sex on drug effect to ensure effective pharmacotherapy.

## 1. Introduction

Although it is widely appreciated that variability in drug performance is governed by a multitude of factors including genetics, age, race, and disease states, the impact of sex has traditionally been under-evaluated. As a result, women have been found to be 1.5 to 1.7 times more likely to develop a drug side effect when compared with their male counterparts [1,2]. Distinct sex-related differences have been demonstrated in the efficacy of cardiovascular pharmacology [3], pain management [4] and cancer immunotherapy [5]. Moreover, the U.S. General Accounting Office (GAO) reported that eight out of the ten drugs withdrawn from the market over a twelve-year period since 1997 were due to greater risks of adverse effects in women [6]. Despite this, pre-clinical research has continued to display a tendency to focus on males in both cell and animal studies [7].

Pharmaceutical excipients are intentionally co-formulated in drug delivery systems to aid the manufacturing processes and drug product performance related to patient acceptability, bioavailability and drug integrity during storage. In particular, excipients are listed as inactive ingredients by the Food and Drug Administration (FDA) who state that their contribution to increasing bioavailability would only be related to their physicochemical (and not physiological) role [8]. However, a growing body of evidence has challenged the inert nature of these agents [9]. For instance, *in vivo* studies demonstrated that sodium acid pyrophosphate [10] and mannitol [11] decreased small intestinal transit time which consequently decreased drug bioavailability by nearly 40%. Similarly, high doses of the solubilising agent polyethylene glycol (PEG) 400 when co-formulated with ranitidine accelerated small intestinal transit and consequently reduced oral bioavailability [12,13,14]. In follow-up studies with low doses of PEG 400, ranitidine bioavailability was increased in both humans [15] and rats [16,17]. This enhancement, however, was limited to males whilst no influence was observed in females [15,16,17].

Sex-specific differences in gastrointestinal physiology, such as the differential expression of membrane transporters, have been suggested to affect drug absorption and drug metabolism [18,19,20]. We have previously shown that the efflux transporter P-glycoprotein (P-gp) was responsible for mediating the variability in drug bioavailability between the sexes via its sex-specific interactions with the excipient PEG 400 [15,16,17,21]. In light of such a significant sex-specific modulation of drug bioavailability by PEG 400 and the scarcity of studies on the influence of sex on excipient behaviour, it is important to establish if other solubilising agents commonly used in drug formulations also exhibit different influences in bioavailability in males and females. As oral administration remains the most patient-acceptable route of drug delivery, and as many drugs currently available in the market are poorly water-soluble, solubilising agents are often included as excipients in drug formulations to improve oral bioavailability [22].

**Figure 1:**
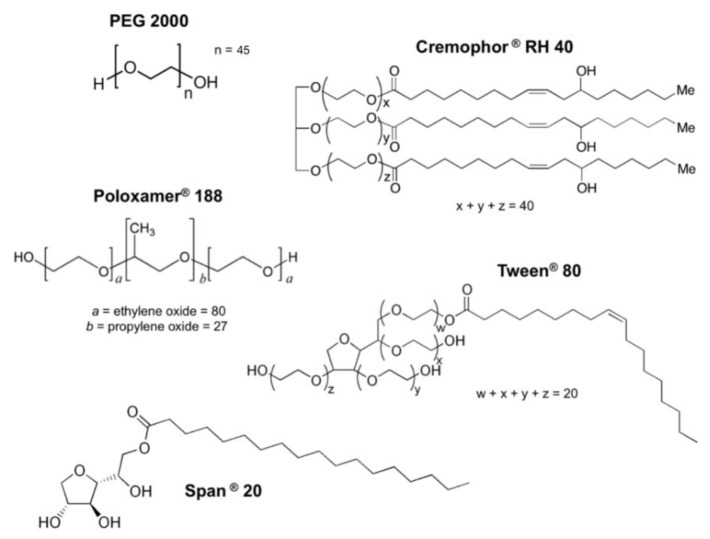
Chemical structures of tested excipients.

The aim of the work was, therefore, to investigate potential sex-specific influences of other solubilising excipients and specifically, polyoxyethylated excipients; polyethylene glycol 2000 (PEG 2000), Cremophor RH 40, Poloxamer 188, Tween 80; and a non-polyoxyethylated excipient, Span 20 (Figure 1). Precisely, their influence on; i) the in vivo bioavailability and in vitro intestinal permeation of the drug ranitidine, a P-gp substrate, and ii) the protein and gene expression of the efflux transporter P-gp in male and female rat models was determined.

## 2. Materials and Methods

### 2.1. Reagents and Materials

Ranitidine hydrochloride, glacial acetic acid, and sodium acetate trihydrate were obtained from Sigma Aldrich (Dorset, UK). Polyethylene glycol 2000 (PEG 2000, Mw: 2000 g/mol), Span 20 (Mw: 346 g/mol) and Tween 80 (Mw: 1310 g/mol) were purchased from Fluka (Dietikon, Switzerland). Poloxamer 188 (Mw: 4600 g/mol) and Cremophor RH 40 (Mw: 4600 g/mol) was from Agenda (Bradford, UK) and BASF (Cheadle, Germany), respectively. Water and acetonitrile were purchased from Fisher Scientific (Loughborough, UK) and were of HPLC grade. All other chemicals and kits are noted individually in the following methods.

### 2.2. Animals

Male and female Wistar rats (8 weeks old, 250 ± 20 g) were used from Harlan UK Ltd. (Oxfordshire, UK).

All animal work was conducted in accordance with the project licence (8002536), approved by the Home Office under the Animals (Scientific Procedures) Act 1986 on 7 June 2012. The rats were housed at room temperature (25 °C) and in a light-dark cycle of 12 h. They were caged in groups of six, allowed to move freely and provided with food and water before the experiment. The day before the experiment, the rats were fasted overnight and individually housed in metabolic cages.

### 2.3. Investigation of the Influence of Excipients on the in Vitro Permeability of Ranitidine

#### 2.3.1. Tissue Preparation

On the day of the experiment, rats were sacrificed with a CO_2_ euthanasia chamber (Schedule 1 method) and the intestine was rapidly removed. In this study, the jejunum was the only intestinal segment assessed as it is the main site of absorption for orally administered drugs. In addition, previous studies confirmed that the jejunum is the optimal intestinal segment to reflect the in vivo influence of excipients specifically on ranitidine absorption [15,16]. The jejunum (10 cm from the ligament of Treitz) was cut, washed with cold KBR solution and put into beakers with Krebs-Bicarbonate Ringer’s (KBR) solution on ice. This consisted of 10 mM d-glucose, 1.2 mM calcium chloride (CaCl_2_), 1.2 mM magnesium chloride (MgCl_2_), 115 mM sodium chloride (NaCl), 25 mM sodium bicarbonate (NaHCO_3_), 0.4 mM monopotassium phosphate (KH_2_PO_4_), 2.4 mM dipotassium phosphate (K_2_HPO_4_), pH 7.4 adjusted with sodium hydroxide/hydrochloric acid [23]. The tissue was allowed to rest for approximately 20 min to achieve a low tissue temperature and thus, minimise potential tissue damage during preparation. About 2–3 cm long pieces from the proximal part of the jejunum were opened along their mesenteric border. The jejunum segments were gently washed with KBR buffer to remove their contents. To obtain the mucosal tissue, sections were placed on an ice-cold glass plate and the serosa was gently squeezed out with tweezers.

#### 2.3.2. Ussing Chamber Set-Up

Once the mucosal tissues were prepared, they were mounted on the vertical Ussing Chamber (Harvard Apparatus Inc., Holliston, MA, USA) as flat sheets on a 0.32 cm^2^ segment holder with needles for stabilisation. 5 mL KBR solution was added to each compartment of the Ussing Chamber and the solutions were gassed with an O_2_/CO_2_ (95%/5%) gas mixture. The chambers were tightly screwed and the entire assembly was controlled at 37 °C.

To evaluate tissue integrity during experiments, tissue transepithelial electrical resistance (TEER) was measured using an EVOMX meter (World Precision Instruments Inc., WPI, Hertfordshire, UK). Any jejunum tissue that showed a TEER value lower than 40 Ω·cm^2^ at the beginning of the experiment was regarded as poorly viable and was excluded. Whenever TEER values decreased by more than 15% from the original value (measured at the end of the 20–30 min equilibration period), the tissue was considered not to be viable and eliminated from the investigation [24].

#### 2.3.3. Transport Study

After an equilibrium period, the experiment was initiated by replacing the blank KBR solution in the donor compartment with pre-warmed ranitidine solution with and without pharmaceutical excipients. 100 μL of the receiver solution was taken to determine the drug concentration by HPLC every 30 min and replaced with an equal volume of a heated blank KBR solution. The receiver solution samples were kept at 4 °C until analysed for drug concentration by HPLC as described in Section 2.5.

Previous experiments in our laboratory using PEG 400 showed that 0.5% *w*/*v* concentrations (12.5 µmol/mL) resulted in the highest enhancement of ranitidine intestinal permeability in the rat jejunum [25]. The same molarity concentrations were therefore used for the other excipients (PEG 2000, Cremophor RH 40, Poloxamer 188, Tween 80 and Span 20) examined in this study.

#### 2.3.4. Calculation

The apparent permeability coefficient (P_app_) in each experiment, in cm/s, was calculated using the following equation:

$$\mathrm{Papp}=\frac{Q}{C\;\times \;A\;\times \;t}$$

where: *Q* (μmol) is the total amount of drug that permeated to the receiver compartment throughout the incubation time; *C* (μmol/mL) is the initial drug concentration in the donor side; *A* (cm^2^) is the diffusion area of the Ussing Chamber and; *t* (s) is the duration of experiment.

### 2.4. Pharmacokinetic Experiments

Each rat was weighed on the day of the experiment and orally administered ranitidine solution with or without a pharmaceutical excipient (PEG 2000 or Cremophor RH 40 or Poloxamer 188 or Tween 80 or Span 20) using an oral gavage needle. The dose of each excipient was 64 µmol/kg and is the same dose that caused the greatest increase in ranitidine bioavailability in a previous Wistar rats’ experiment with PEG 400 [16]. Comparable excipient concentrations were used in the in vitro and in vivo studies, taking the animal weight and administration volume into account.

Following oral administration, approximately 250–300 μL of blood was collected from the rats’ tail vein into anticoagulant centrifuge tubes (BD Microtainer^®^ K2E Becton, Dickinson and Company, Franklin Lakes, NJ, USA) at 0.5, 1.25, 2, 3, 4, 6, and 8 h. The rats were killed in a CO_2_ euthanasia chamber and about 1 mL of blood was taken by cardiac puncture. All blood samples were centrifuged at 10,000 rpm for 10 min on a Centrifuge 5804R (Eppendorf AG, Hamburg, Germany) within 8 h of collection. 50 µL of the supernatant was collected and placed into a 1.5 mL Eppendorf tube. The same volume of acetonitrile was added to precipitate the plasma proteins. The solution was vortex-mixed for 1 min to which 100 µL HPLC grade water was subsequently added. After vortex-mixing for another minute, the samples were centrifuged at 4 °C for 10 min at 10,000 rpm. The supernatant was collected and kept at −20 °C until analysis [16].

### 2.5. Methods of Analysis

The samples were stored at −20 °C until analysis and allowed to thaw at room temperature before processing. The sample was subjected to HPLC-UV assay using a previously validated method [26]. The column used was a 5 µm Luna SCX (Phenomenex, UK); the mobile phase was a mixture of 20:80 (acetonitrile):(0.1M sodium acetate pH = 5.0) with a flow rate of 2 mL/min and 40 µL of injection volume.

### 2.6. Pharmacokinetic Analysis

Pharmacokinetic parameters, involving *C*_max_, *t*_max_, AUC_0–480_ and AUC_0–infinity_ was calculated by non-compartmental analyses using a free Microsoft Excel add-in, PKSolver [27].

### 2.7. Western Blot Analysis to Determine the Influence of Excipients on P-gp Protein Expression

#### 2.7.1. Animal Treatment

On the day of the experiment, each rat was weighed and orally administered with a solution of one of the pharmaceutical excipients. Doses were the same as those used in Section 2.4. After 90 min, the rats were sacrificed in a CO_2_ euthanasia chamber. Then the intestines were rapidly removed and the jejunal segments were prepared as described in Section 2.3.1.

90 min post dosing was selected following a previous on the influence of time using PEG 400 as the model excipient. P-gp expression quantification had been after 15 min, 30 min, 60 min, 90 min, 120 min or 180 min post-dosing. The results showed a peak effect at 90 min post PEG dosing [17].

#### 2.7.2. Sample Preparation

The mucosal tissues (about 60 mg) were cut into small pieces and homogenised in 3 mL lysis buffer (freshly prepared with 50 mM Tris, 250 mM NaCl, 5 mM ethylenediaminetetraacetic acid (EDTA), 1 mM sodium orthovanadate (Na_3_VO_4_), 1 mM phenylmethysulfonyl fluoride (PMSF), 1% octylphenoxy poly(ethyleneoxy) ethanol (Nonidet P40) and protease inhibitor cocktail in phosphate buffered saline (PBS)) at 10,000 rpm for 20 s on ice with a T18 digital ULTRA-TURRAX^®^ (IKA, Wilmington, CA, USA). The tissue homogenates were incubated at 4 °C for 2 h and then centrifuged at 10,000 rpm for 10 min. The total tissue protein was collected in the supernatants and its concentration was subsequently determined with the Pierce™ BCA Assay Protein kit (ThermoFisher, Loughborough, UK) according to the manufacturer’s instructions.

#### 2.7.3. Protein Analysis

To measure the targeted P-gp level, samples containing 25 μg total protein were suspended in lithium dodecyl sulfate (LDS) sample loading buffer (Invitrogen, Carlsbad, CA, USA) and denatured for 10 min at 70 °C. As a molecular weight marker, 5 μL of Sharp Pre-Stained protein standard (Invitrogen) was loaded on each gel.

Proteins in the samples were separated by electrophoresis in a NuPAGE™ Novex™ 4–12% Bis-Tris gel (Invitrogen) and transferred to a nitrocellulose membrane with XCell SureLock™ Mini-Cell Electrophoresis System (Invitrogen) according to the manufacturer’s instructions. Nitrocellulose membranes were blocked with 3% bovine serum albumin (BSA) in TBS-T (0.1% Tween 20 in tris-buffered saline) and incubated for 1 h at room temperature. For the detection of P-gp and reference protein (anti-beta-actin), blots were incubated for 1 h at room temperature with the respective primary antibodies diluted in 3% BSA in TBS-T: mouse monoclonal anti-P-gp (C-219 3:200; Enzo Life Science, Exeter, UK) and anti-β-actin mouse monoclonal antibody (1:2000; Sigma-Aldrich, Poole, UK). Bound antibodies were detected with affinity-purified rabbit anti-mouse IgG coupled to horseradish peroxidase (secondary antibody; Sigma-Aldrich, Dorset, UK) diluted 1:5000 in 3% BSA in TBS-T.

After 1 h incubation with the secondary antibody conjugated with horseradish peroxidase, protein bands were visualised by chemiluminescence detection with Pierce™ ECL Western Blotting Substrate (ThermoFisher, Loughborough, UK), and subsequently photographed with a ChemiDoc XRS camera (Bio-Rad, Hertfordshire, UK). P-gp and reference protein bands were qualified using the Image Lab™ software (Bio-Rad, Hertfordshire, UK). To calculate the relative P-gp contents in the different samples, the reference protein band in each sample was set to 1 and the intensity of the P-gp band was measured relative to it.

### 2.8. Real-Time Reverse-Transcription Polymerase Chain Reaction to Determine the Influence of Excipients on the Expression of P-gp Gene

#### 2.8.1. RNA Isolation

Following the collection of mucosal tissues (as described in Section 2.7.1), the tissues were kept in RNA later^®^ Stabilization Solution (Thermofisher, Loughborough, UK). Total RNA in each intestinal sample was isolated and purified with PureLink^®^ RNA Mini Kit (Thermofisher, Loughborough, UK). RNA concentration was measured with Nanodrop 2000 (Thermofisher, Loughborough, UK) according to the manufacturer’s instructions.

#### 2.8.2. RNA Level Analysis

Subsequently, the quantification of the target RNA was conducted as follows; 1 mg total RNA of each sample was reverse transcribed using the iScript™ cDNA Synthesis Kit (Bio-Rad, Hertfordshire, UK). To quantify the amount of P-gp mRNA (*mdr1a* and *mdr1b*), real-time PCR was performed on the 7500 Real Time PCR System (Applied Biosystems, Thermofisher, Loughborough, UK) using the method described in the study by MacLean et al. [28] 50 μL of the PCR reaction contained 25 μL of PowerUp™ SYBR Green PCR Master Mix (Thermofisher, Loughborough, UK), 500 nM each of forward and reverse primers, and 1 μg of cDNA. Anti-beta actin was used for the normalisation and amplification of 1 μg cDNA respectively. Real-time PCR was carried out in 96 well PCR plates (Thermofisher, Loughborough, UK). The amplification program for all genes consisted of one pre-incubation cycle at 95 °C with a 10 min hold, followed by 45 amplification cycles with denaturation at 95 °C with a 10 s hold, an annealing temperature of 50 °C with a 10 s hold and an extension at 72 °C with a 10 s hold. Amplification was followed by a melting curve analysis which ran for one cycle with denaturation at 95 °C with a 1 s hold, annealing at 65 °C with a 15 s hold, and melting at 95 °C with a 1 s hold. Distilled water was included as a negative control in each run to access specificity of primers and possible contaminants.

Primers (shown in Table 1) were designed by primer-BLAST searching with publicly available sequence information of the GeneBank of the National Center for Biotechnology Information (NCBI) and purchased from Eurofins (Eurofins Genomics, Germany).

#### 2.8.3. Data Analysis

Relative expression of *mdr1a* and *mdr1b* mRNA in different intestinal segments were calculated using the 7500 software (version 2.0.6, Thermofisher, Loughborough, UK). The average of the threshold cycle (Ct) values for tested genes (*mdr1a* and *mdr1b*) and the internal control (anti-beta actin) was taken. The differences between Ct values for tested genes and internal control (∆Ct) were then calculated for all the experimental samples.

### 2.9. Statistical Analysis

All results are expressed as mean ± SD (*n* = 6). The control and test group data were analysed by one-way ANOVA, followed by post-hoc Tukey analysis with a 95% confidence interval using IBM SPSS Statistics 16 (SPSS Inc., Chicago, Illinois, USA). A minimum *p*-value of 0.05 was used as the significance level for the tests.

## 3. Results

### 3.1. In Vitro Intestinal Permeability

**Figure 2:**
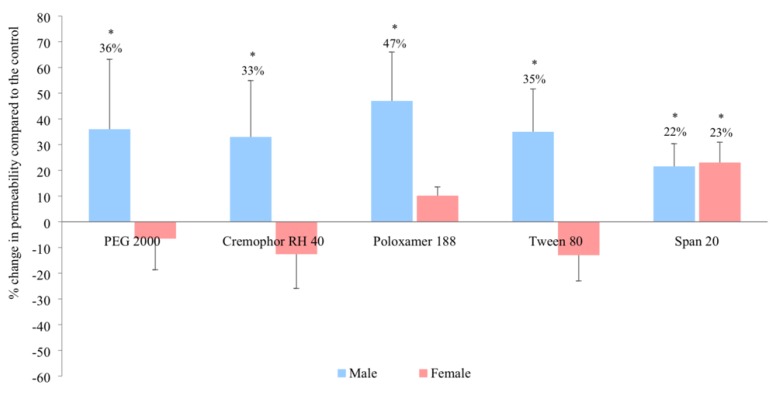
Percentage change in in vitro ranitidine permeability in the rat jejunum in the presence of the different excipients in males and females (mean ± SD, *n* = 6). * Values are statistically different when compared to the control (i.e., no excipient) and the excipient groups at *p* < 0.05.

In the male rat jejunum, the in vitro intestinal permeability of ranitidine increased in the presence of PEG 2000, Cremophor RH 40, Poloaxamer 188 and Tween 80 by 36%, 33%, 47% and 35% respectively (Figure 2). In contrast, no enhancement was observed in female rats in the presence of these excipients when compared with the control. A distinctly different result was observed for Span 20 where ranitidine permeability significantly increased in both male and female rats by 22% and 23% respectively (*p* < 0.05).

### 3.2. In Vivo Bioavailability

**Figure 3:**
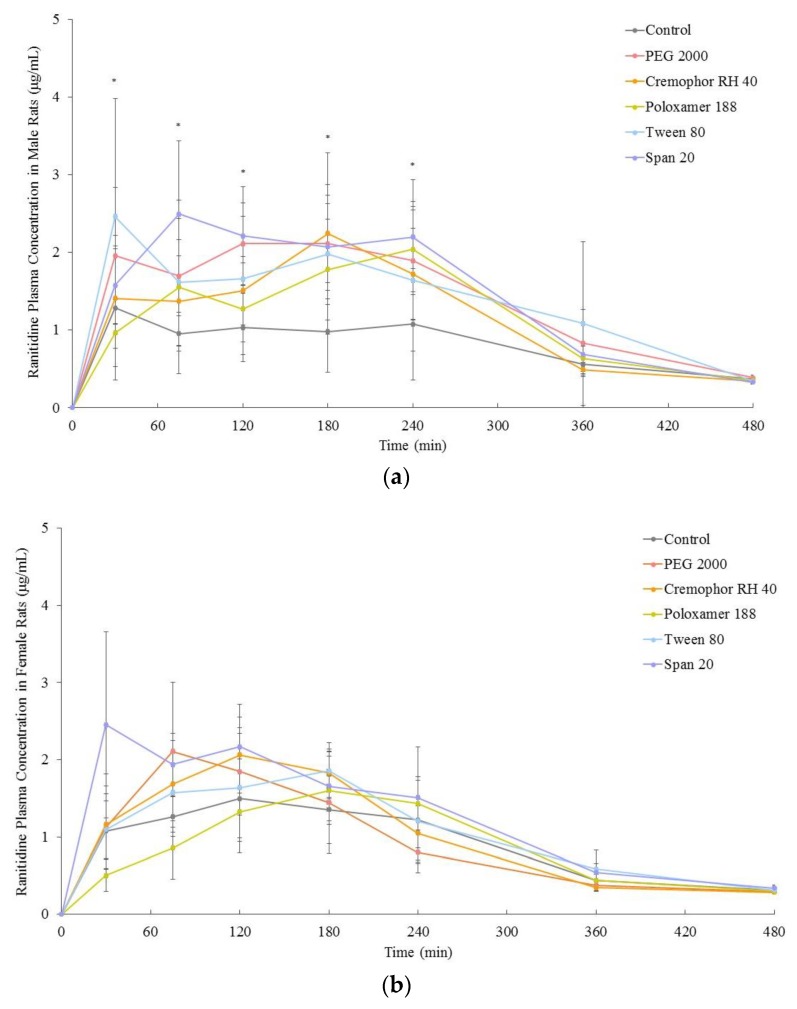
In vivo mean plasma concentration-time curve of ranitidine in the absence (control) or presence of excipients in male **(a)** and female **(b)** rats (*n* = 6).

**Figure 4:**
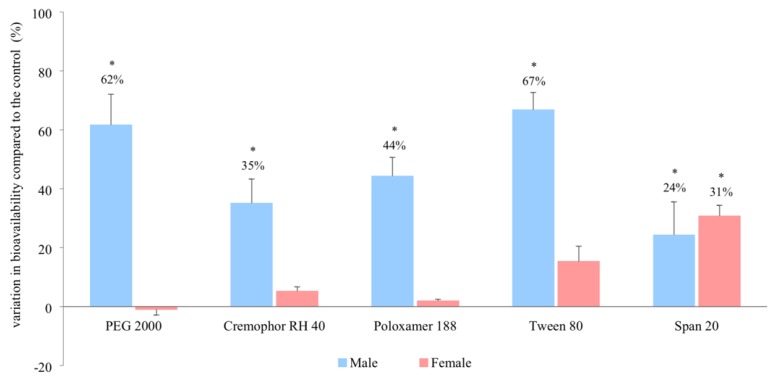
Percentage change in ranitidine bioavailability (AUC_0–infinity_) in the presence of excipients in male and female Wistar rats (mean ± SD, *n* = 6). * Values are statistically different between the control (i.e., no excipient) and the excipient groups at *p* < 0.05.

The ranitidine plasma concentration–time profiles in the presence and absence of these excipients for males and females are shown in Figure 3. The *in vivo* bioavailability of ranitidine (area under the curve, 0-infinity) was significantly increased in male rats in the presence of PEG 2000, Cremophor RH 40, Poloxamer 188 and Tween 80 by 62%, 35%, 44%, and 67% respectively whilst no significant changes were observed in female rats (Figure 4). The co-formulation of ranitidine and Span 20, however, significantly increased drug bioavailability in both males and females by 24% and 31% respectively when compared with the controls (Figure 4 and Table 2).

### 3.3. Relative P-glycoprotein Abundance

**Figure 5:**
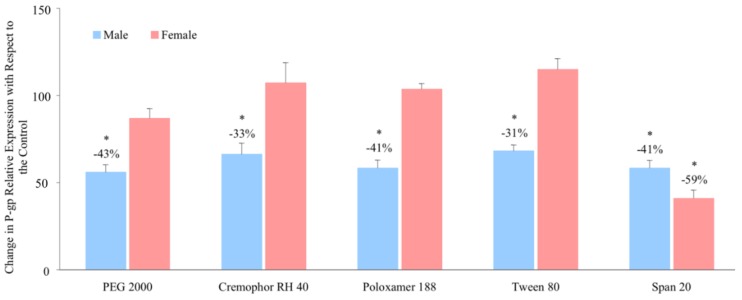
Percentage change in P-gp protein level in the presence of excipients in male and female Wistar rats (mean ± SD, *n* = 6). * Values are statistically different between the control (i.e, no excipient) and the excipient groups at *p* < 0.05.

With regards to P-gp protein abundance, all the solubilising excipients decreased the intestinal expression of P-gp by 31–43% in male rats. No significant observations were demonstrated in the intestinal expression of P-gp in female rats, except in the presence of Span 20 where protein levels decreased by 59% when compared with the control (Figure 5).

### 3.4. P-glycoprotein Gene Expression

**Figure 6:**
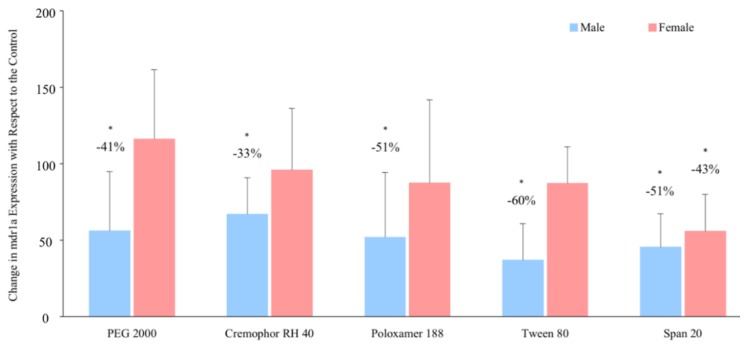
Percentage changes in *mdr1a* mRNA expression in the presence of excipients in male and female Wistar rats (mean ± SD, *n* = 6). * Values are statistically different between the control (i.e., no excipient) and the excipient groups at *p* < 0.05.

In terms of P-gp gene expression, the solubilising excipients reduced *mdr1a* mRNA expression in the presence of PEG 2000, Cremophor RH 40, Poloxamer 188 and Tween 80 by 33 – 60% in male rats but had no influence in females. In the presence of Span 20, however, *mdr1a* expression in both male and female rats significantly reduced by 51% and 43% respectively (Figure 6). No obvious trend was observed in *mdr1b* mRNA expression in males and females in the presence of these excipients (Supplementary Figure S1).

## 4. Discussion

Our results add to the growing body of evidence that challenges the ‘inert’ nature of excipients. In addition, we show that the influence of certain excipients is subject to the sex of the species. The polyoxyethylated excipients, PEG 2000, Cremophor RH 40, Poloxamer 188 and Tween 80 all increased the in vivo bioavailability of ranitidine in male but not in female rats. The differential increase in in vivo bioavailability between the sexes (Figure 3 and Figure 4) was reflected in the in vitro permeability of ranitidine due to the presence of these excipients (Figure 2).

Moreover, the excipients significantly increased ranitidine in vitro jejunal permeability and *in vivo* bioavailability in males and this was reflected in their influence on the relative P-gp level expressed; all the excipients exhibited inhibitory influences on the activity and expression of P-gp. Interestingly, in females, the excipients did not change relative P-gp expression (Figure 5 and Figure 6).

In contrast, Span 20, a non-polyoxyethylated excipient, enhanced in vitro and in vivo bioavailability in both males and females showing no sex differences. Consequently, the enhancement of drug bioavailability in males may be related to the polyoxyethylated structures of PEG 2000, Cremophor RH 40, Poloxamer 188 and Tween 80.

In agreement with our previous study on PEG 400 [18], PEG 2000 is a modulator of the efflux transporter P-gp, although this effect is limited to male rats. PEGs can inhibit the efflux transport of P-gp to increase the bioavailability of P-gp substrates. For example, PEG 2000 and PEG 20,000 reduce the secretory transport of P-gp-mediated Rhodamine 123 in male rats [29,30]. Our study, however, confirms that PEG 2000 is capable of modifying the activity and expression of P-gp to enhance the bioavailability of P-gp-mediated drugs in a sex-dependent manner. With regards to Cremophor RH 40, its effect on the P-gp substrate digoxin was to significantly increase its bioavailability by 22% in males [31]. In the case of Tween 80, Rege *et al.* reported that the permeability of Rhodamine 123 (a P-gp substrate) was increased from the apical-to-basolateral membrane but decreased from the basolateral-to-apical membrane in Caco-2 cell monolayers in the presence of the excipient [32]. At concentrations below 0.01% (*w*/*v*), Tween 80 was able to decrease the expression of P-gp which resulted to an enhanced uptake of P-gp-mediated Rhodamine 123 [33]. In addition, this study is the first to describe the in vivo effects of Tween 80 on the function of P-gp with in vitro results strongly proving its effects. However, herein, Tween 80 not only proved its active interaction with P-gp but also demonstrated its sex-dependent interaction on P-gp function.

The underlying mechanism behind this sex-based phenomenon is unclear although a number of hypotheses are possible. Firstly, an innately higher intestinal P-gp expression was reported in male rodents and male human species when compared with females [34,35]. This, however, cannot be considered as the sole reason for sex-differences as variances in absorption is only reflected in some P-gp substrates even in the absence of studied excipients. Secondly, surfactants have been shown to modulate P-gp activity via altering the fluid environment of the lipid cell membrane. This consequently led to a reduction of ATPase activity as well as a decrease in affinity of P-gp for ATP in conjunction with a depletion of intracellular ATP [29]. The differences of P-gp ATPase activity between males and females are unknown, however, all tested excipients, including Span 20, have been reported to alter the ATPase activity of P-gp. This can also influence its efflux protein transporter function on the intestinal membrane [36,37,38,39]. If P-gp ATPase activity differed between males and females, a sex-related difference should have been seen in the intestinal absorption of ranitidine in the presence of Span 20. Moreover, several other studies have further shown that non-ionic surfactants are effective at modifying P-gp ATPase activity at concentrations lower than their critical micelle concentration (CMC). This inhibitory effect, however, is decreased at or above the CMC value of the surfactant due to micelle formation [40,41]. As all excipient doses used in this study were higher than their CMC (Cremophor RH 40, 0.01% [42]; Poloxamer 188, 0.2% [43]; Tween 80, 0.0014% [44]; and Span 20, 0.07% [45], the sex-related modification on the P-gp ATPase activity is less likely to be a plausible reason behind this phenomenon.

Interestingly, the alteration in membrane lipid environment can also lead to changes in P-gp protein expression. P-gp is highly sensitive to the lipid environment; the membrane lipid bilayer can consequently be affected in the presence of excipients such as PEG 2000, Cremophor RH 40, Poloxamer 188 and Tween 80 in this study which all contain hydrophilic ethylene oxide (EO) units. The interaction between excipients and P-gp in the lipid membrane is due to transient hydrogen bond formation of EO groups with hydrogen bond donor groups of the transmembrane domains of P-gp [46]. As such, the EO unit can be inserted into the polar head group regions of the cell membrane [47,48,49] and elicit a change in the lipid environment. Lastly, female rat intestinal P-gp has been found to be less sensitive to pharmaceutical excipients than in males [18]. Based on the aforementioned findings, the authors have suggested that this may be due to the female intestinal membrane being more resilient to changes in fluidity and/or undergoing slower epithelial renewal rates than their male counterparts.

The expression of P-gp was also recently reported to be linked to a group of nuclear receptors such as the nuclear receptor proteins retinoic acid receptor (RAR), farnesoid receptor (FXR), steroid-activated receptor (SXR) and pregnane receptor (PXR, in rodents) which have lipid ligands [50,51,52]. The ability of nuclear receptors to cross-react with the protein binding sites and to govern similar membrane regulatory cascades indicates a bi-directional transcriptional regulatory pathway between lipids and P-gp [53,54,55]. However, the interaction between excipients and nuclear receptors and the qualification of the nuclear receptors are unknown. Therefore, the excipients studied in this paper may interact with the receptor pathways, resulting in the observed reduction in P-gp expression in a similar manner to previously reported lipid agents, Peceol^®^ and Gelucire^®^ 44/14 [56].

## 5. Conclusions

In this study, we identified that a number of solubilising agents including PEG 2000, Cremophor RH 40, Poloxamer 188 and Tween 80 were capable of reducing the activity and expression of intestinal P-gp, resulting in the enhancement in ranitidine bioavailability in male rats, but not in females. In contrast, Span 20 enhanced bioavailability in both male and female rats in a non-sex-dependent manner. The underlying mechanism for the sex-related influence of excipients on transporters may be related to the chemical structure of the excipients since all the tested excipients, bar Span 20, are polyoxyethylated. The sex differences demonstrated by this study highlights the need for the screening of ‘inert’ pharmaceutical excipients in terms of their selection and use for oral drug development. In particular, the results reported here are of clinical importance for P-gp drug substrates.

## Figures and Tables

**Table 1:** Primers used for the analysis of P-gp gene expression by real-time qPCR.

Gene	Primer (5′ – 3′)		Amplicon (bp)	GenBank^®^ Accession
mdr1a	Forward	CACCATCCAGAACGCAGACT	159	NM_133401
	Reverse	ACATCTCGCATGGTCACAGTT		
mdr1b	Forward	AACGCAGACTTGATCGTGGT	144	NM_012623
	Reverse	AGCACCTCAAATACTCCCAGC		
anti-beta actin	Forward	GCAGGAGTACGATGAGTCCG	74	NM_031144
	Reverse	ACGCAGCTCAGTAACAGTCC		

**Table 2:** Effect of excipients on the pharmacokinetic parameters of ranitidine in male and female Wistar rats (mean ± SD, *n* = 6). * Values are statistically different between the control and excipient groups at *p* < 0.05.

Pharmacokinetic Parameters	Control (i.e., No Excipient)	PEG 2000	Cremophor RH 40	Poloxamer 188	Tween 80	Span 20
**Male**						
AUC_0–480_ (*μg.min/mL*)	391 ± 108	680 ± 167 *	563 ± 94 *	561 ± 112 *	670 ± 146 *	546 ± 80 *
AUC_0–infinity/∞_ (*μg.min/mL*)	485 ± 109	784 ± 171 *	655 ± 85 *	700 ± 77 *	809 ± 212 *	603 ± 91 *
*c*_max_ (*μg/mL*)	2.0 ± 0.8	3.5 ± 0.7 *	3.4 ± 0.6 *	3.9 ± 0.7 *	3.8 ± 0.9 *	3.8 ± 0.5 *
*t*_max_ (*min*)	146 ± 110	150 ± 79	155 ± 61	195 ± 72	160 ± 128	157 ± 95
**Female**						
AUC_0–480_ (*μg.min/mL*)	437 ± 59	454 ± 74	490 ± 27	422 ± 61	507 ± 33	613 ± 76 *
AUC_0–infinity/∞_ (*μg.min/mL*)	517 ± 47	512 ± 66	544 ± 26	527 ± 61	596 ± 52	676 ± 79 *
*c*_max_ (*μg/mL*)	2.5 ± 0.2	1.8 ± 0.7	2.1 ± 0.3	1.9 ± 0.5	2.6 ± 0.4	3.2 ± 0.6
*t*_max_ (*min*)	127 ± 75	107 ± 42	98 ± 38	190 ± 45	150 ± 35	70 ± 65

## Supplementary Materials

The following are available online at https://www.mdpi.com/1999-4923/11/5/228/s1, Figure S1: Percentage changes in *mdr1b* mRNA expression in the presence of excipients in male and female Wistar rats (mean ± SD, *n* = 6).
