# Supplementary material for: Sex-Dependence in the Effect of Pharmaceutical Excipients: Polyoxyethylated Solubilising Excipients Increase Oral Drug Bioavailability in Male but Not Female Rats
Source: Pharmaceutics. 2019 May 10;11(5):228. doi: 10.3390/pharmaceutics11050228 (PMC6571596; doi:10.3390/pharmaceutics11050228)
Supplement: Supplementary file 1 [file pharmaceutics-11-00228-s001.pdf]

pharmaceutics

Supplementary Materials: Sex-Dependence in the Effect of Pharmaceutical Excipients: Polyoxyethylated Solubilising Excipients Increase Oral Drug Bioavailability in Male but not Female Rats

Yang Mai, Liu Dou, Christine M. Madla, Sudaxshina Murdan and Abdul W. Basit

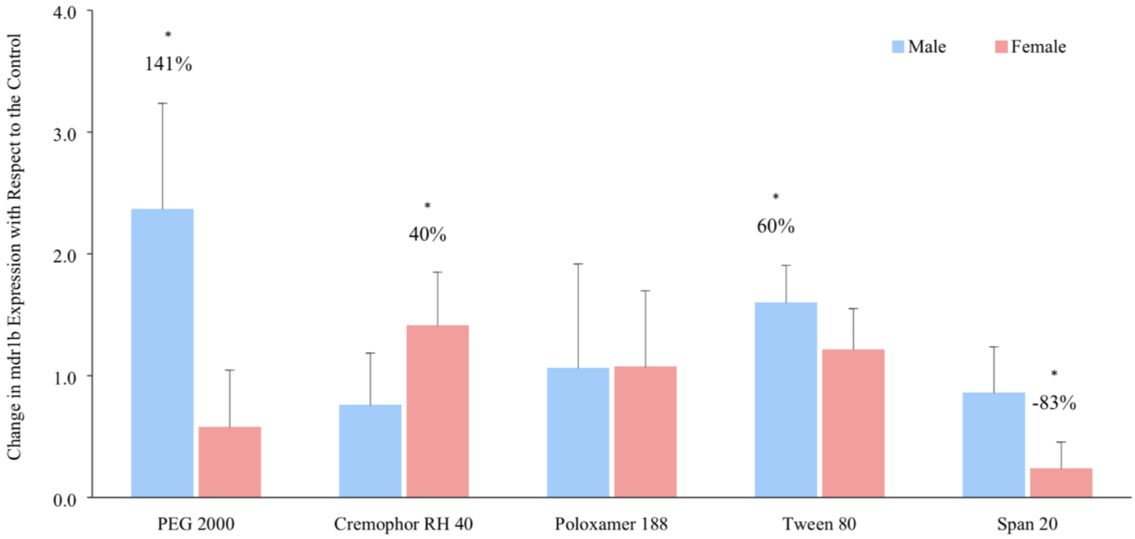

Figure S1. Percentage changes in *mdr1b* mRNA expression in the presence of excipients in male and female Wistar rats (mean ± SD, $n = 6$).
